# Von Willebrand factor processing in patients with advanced chronic liver disease and its relation to portal hypertension and clinical outcome

**DOI:** 10.1007/s12072-023-10577-y

**Published:** 2023-08-21

**Authors:** Benedikt Simbrunner, Ida Falk Villesen, Bernhard Scheiner, Rafael Paternostro, Philipp Schwabl, Albert Friedrich Stättermayer, Rodrig Marculescu, Matthias Pinter, Peter Quehenberger, Michael Trauner, Morten Karsdal, Ton Lisman, Thomas Reiberger, Diana Julie Leeming, Mattias Mandorfer

**Affiliations:** 1 Division of Gastroenterology and Hepatology, Department of Internal Medicine III, Medical University of Vienna, Waehringer Guertel 18-20, 1090 Vienna, Austria; 2 Vienna Hepatic Hemodynamic Lab, Division of Gastroenterology and Hepatology, Medical University of Vienna, Vienna, Austria; 3 Christian Doppler Laboratory for Portal Hypertension and Liver Fibrosis, Medical University of Vienna, Vienna, Austria; 4 Ludwig Boltzmann Institute for Rare and Undiagnosed Diseases (LBI-RUD), Vienna, Austria; 5 CeMM Research Center for Molecular Medicine of the Austrian Academy of Sciences, Vienna, Austria; 6 Nordic Bioscience, Herlev, Denmark; 7 University of Copenhagen, Copenhagen, Denmark; 8 Department of Laboratory Medicine, Medical University of Vienna, Vienna, Austria; 9 Surgical Research Laboratory, Department of Surgery, University of Groningen, University Medical Centre Groningen, Groningen, The Netherlands

**Keywords:** Cirrhosis, CSPH, VWF, Propeptide, ADAMTS13, Endothelial dysfunction

## Abstract

**Background and aims:**

Endothelial dysfunction and portal hypertension (PH) are reflected by increased von Willebrand factor antigen (VWF-Ag) levels in advanced chronic liver disease (ACLD). This study investigated VWF release and cleavage and their association with PH and clinical outcomes.

**Methods:**

Levels of VWF-Ag, VWF-N (VWF-propeptide), and VWF-A (VWF processed by the main VWF-cleaving protease ADAMTS13) were assessed in 229 patients with clinically stable ACLD (hepatic venous pressure gradient [HVPG] ≥ 6 mmHg; absence of bacterial infections or acute decompensation) undergoing HVPG-measurement. Liver-healthy individuals served as controls (n = 24).

**Results:**

VWF-Ag and VWF-N were similarly accurate for the identification of clinically significant PH (CSPH; HVPG ≥ 10 mmHg) in compensated ACLD (AUROC: VWF-Ag 0.748; VWF-N 0.728). ADAMTS13 activity was similar between patients with ACLD and controls and did not correlate with PH and disease severity, whereas VWF cleavage decreased in patients with CSPH (i.e., VWF-Ag/-A-ratio increased). In vitro VWF activity strongly reflected VWF-Ag levels (Spearman’s r = 0.874, p < 0.001), but decreased (vs. controls) in patients with CSPH when normalized to VWF-Ag levels (VWF-activity/-Ag-ratio). VWF-Act/-Ag ratio correlated negatively with ADAMTS13 activity (r =– 0.256, p < 0.001). ADAMTS13 activity was independently predictive for (i) portal vein thrombosis (PVT) and (ii) hepatic decompensation or liver-related death.

**Conclusions:**

VWF-Ag levels and its propeptide are similarly suitable surrogates of PH in patients with compensated ACLD. ADAMTS13-Act was not linked to disease and PH severity, however, when normalized to VWF-Ag, both VWF cleavage and VWF activity were decreased in patients with CSPH, as compared to liver-healthy individuals. Low ADAMTS13-Act was associated with presumably more procoagulant VWF and adverse outcomes.

**Clinical trial number:**

NCT03267615

**Supplementary Information:**

The online version contains supplementary material available at 10.1007/s12072-023-10577-y.

## Introduction

Portal hypertension (PH) is a key feature of advanced chronic liver disease (ACLD) that strongly determines the development of liver-related complications and mortality and may be quantified by minimally invasive measurement of hepatic venous pressure gradient (HVPG) [1]. Experimental studies indicate that persistent perturbation of liver sinusoidal endothelial cells (LSEC; i.e., endothelial dysfunction) leads to activation of hepatic stellate cells (HSC) and intrahepatic vasoconstriction [2], thereby contributing to PH.

von Willebrand factor (VWF) is released by activated endothelial cells and plays a decisive role in hemostasis by mediating the adhesion of platelets to subendothelial collagen and other platelet-adhesive proteins that are exposed during vascular injury [3]. Early studies have found that VWF is elevated in patients with ACLD and linked this observation to endothelial dysfunction, which seemed to be related to endotoxemia [4–6] arising from pathological bacterial translocation. Importantly, VWF antigen (VWF-Ag) also exhibited diagnostic value for clinically significant PH (CSPH; defined by an HVPG ≥ 10 mmHg [7]) as well as prognostic value for disease progression [8–12], even after etiological cure [13, 14]. Nevertheless, it remains unclear whether VWF-Ag is the most capable VWF-related non-invasive test for CSPH.

Previous research addressing whether high VWF levels are relevant for the fragile hemostatic balance in patients with ACLD suggested that increased VWF may compensate deficiencies in the number and eventually also function of platelets in ACLD [15]. Importantly, large VWF multimers released from endothelial cells are subjected to cleavage by the protease “a disintegrin and metalloproteinase with a thrombospondin type 1 motif, member 13” (ADAMTS13) [16]. However, there is an ongoing controversy on ADAMTS13 activity and its implications for VWF function and the hemostatic equilibrium of patients with ACLD [17].

Our study aimed to investigate the relation of VWF release and clearance with PH and liver disease severity in a large cohort of patients with ACLD undergoing HVPG measurement, by measuring VWF propeptide (VWF-N; reflecting release from endothelial cells), VWF antigen levels (VWF-Ag), and ADAMTS13-cleaved VWF (VWF-A). Furthermore, we assessed ADAMTS13 activity (ADAMTS13-Act) and in vitro VWF activity (VWF-Act) in a sizable subgroup to determine their association with VWF levels, VWF cleavage, and PH.

## Patients and methods

### Study design and clinical characterization

Between 01/2017 and 06/2020, patients with ACLD (defined by an HVPG ≥ 6 mmHg) undergoing liver vein catheterization at the Medical University of Vienna were included in the prospective Vienna Cirrhosis Study (VICIS). For this study, we excluded patients with pre-/post-hepatic/non-cirrhotic PH, transjugular intrahepatic portosystemic shunt (TIPS), hepatocellular carcinoma beyond Milan criteria and active extrahepatic malignancies, previous liver transplantation, non-selective betablockers, and bacterial infection or non-elective hospitalization at the time of liver vein catheterization (Supplementary figure S1). In the final study cohort of 229 patients, VWF antigen (VWF-Ag) and blood biomarkers of VWF release, and VWF degradation were measured. In a subgroup of 166 patients, ADAMTS13-Act and VWF-Act was assessed. Notably, patients included in this study represent the majority of the study cohort reported in a previous publication that investigated the link between liver fibrogenesis and systemic inflammation, i.e., focusing on a different pathophysiological aspect of ACLD [18]. The patients included in this study have not been analyzed previously in regard to the diagnostic value of VWF biomarkers, i.e., there was no overlap with previous work from our center [10, 11].

### Measurement of hepatic venous pressure gradient

HVPG was measured according to a standard operating procedure, as published previously [19]. Briefly, a catheter introducer sheath was placed in the right internal jugular vein. A balloon catheter was introduced in a large hepatic vein under fluoroscopic guidance. Correct wedge position was verified by injection of contrast media, while the inflated balloon was blocking the outflow of the cannulated hepatic vein. HVPG was assessed by at least three measurements of free and wedged hepatic venous pressure.

### Biomarker measurement

Biomarkers were measured in blood samples obtained via the catheter introducer sheath during HVPG measurement. Measurements were performed by personnel blinded to clinical and hemodynamic patient characteristics. VWF-Ag levels were assessed by a latex agglutination assay (STA LIATEST vWF:Ag, Diagnostica Stago, Asnieres, France) and VWF-Act was measured by turbidimetric VWF:GPIbM assay (Innovance VWF Ac, Siemens, Marburg, Germany) in citrate platelet poor plasma (< 10×10^9^ L^–1^, according to the recommendation of the Clinical and Laboratory Standards Institute [CLSI, Guideline H21-A5]). ADAMTS13 activity was quantified in citrate plasma by a standardized chromogenic enzyme-linked immunosorbent assay (ELISA) determining GST-VWF73 substrate cleavage (Technozym ADAMTS13 Activity, Technoclone, Vienna, Austria). C-reactive protein (CRP), Interleukin-6 (IL-6), procalcitonin (PCT), and lipopolysaccharide binding protein (LBP) were measured and were already reported in an earlier publication from the same patient cohort in a different context [18]. All the above-mentioned parameters as well as routine laboratory tests were measured by the ISO-accredited Department of Laboratory Medicine, Medical University of Vienna, Austria. VWF propeptide (VWF-N; i.e., released N-terminal propeptide of VWF) and ADAMTS13-processed VWF (VWF-A; i.e., neoepitope of ADAMTS13-mediated degradation of VWF) were assessed in EDTA plasma by competitive enzyme-linked immunosorbent assay (ELISA). More detailed information is delineated in the Supplementary material.

### Liver-healthy controls

Twenty-four individuals without liver disease and cardiovascular disease, diabetes, malignancies, and intake of platelet-inhibitors or anticoagulants undergoing blood withdrawal served as controls for the study. VWF biomarkers and ADAMTS13-Act were measured and compared to patients with ACLD.

### Statistical analysis

Statistical analyses were performed using IBM SPSS Statistics 27 (IBM, Armonk, NY, USA) or GraphPad Prism 9 (GraphPad Software, La Jolla, CA, USA). Categorical variables are reported as absolute (n) and relative (%) frequencies. Continuous variables are displayed as mean ± standard error of the mean or median with interquartile range (IQR), as appropriate. Student’s t test, Mann–Whitney U test, analysis of variance (ANOVA), or Kruskal–Wallis test were used for group comparisons of continuous variables, as applicable, and Dunn’s multiple comparisons test was applied for post-hoc analysis. Group comparisons of categorical variables were performed using Chi-squared or Fisher’s exact test. Normal distribution was determined by Shapiro–Wilk and Kolmogorov–Smirnov tests. Correlation between parameters were assessed by Spearman’s correlation coefficients (95% confidence interval). The diagnostic value of VWF biomarkers for prediction of CSPH (HVPG ≥ 10 mmHg) was determined by area-under-the-receiver operating characteristics (AUROC). Optimal cut-off levels were determined by Youden’s index (sensitivity + specificity − 1), followed by assessment of positive (PPV) and negative (NPV) predictive values, and positive (PLR) and negative (NLR) likelihood ratios. The same analyses were performed using highly sensitive and specific (≥ 90%) cut-offs for diagnosing CSPH. Patients were referred to abdominal imaging every 6 months for hepatocellular carcinoma screening. Concordantly, we record any occurrence of PVT from abdominal imaging results as part of a standard operating procedure during routine visits in the cirrhosis outpatient clinic. Cox regression models with backwards elimination were calculated to assess predictors of (i) portal vein thrombosis (PVT) and (ii) the composite endpoint of first/further decompensation or liver-related death, as described previously [20]. The level of significance was set at a two-sided p value < 0.05 for all analyses.

### Compliance with ethical standards

The study was conducted in accordance with the principles of the Declaration of Helsinki and its amendments, was approved by the local ethics committee of the Medical University of Vienna (EK 1262/2017) and included patients of the prospective VICIS study (NCT03267615). All patients provided written informed consent for hepatic vein catheterization and participation in the VICIS study. Liver-healthy individuals gave written informed consent to participate in the VICIS study as liver-healthy controls.

## Results

### Patient characteristics

The study cohort comprised 229 patients with a median age of 58 (50–67) years, 65% male sex (n = 148), and alcohol-related liver disease (ALD; n = 105, 46%) and viral hepatitis (n = 41, 18%) were the most common etiologies. Ninety-two (40%) patients had compensated ACLD, and 201 (88%) patients had CSPH. Median HVPG was 18 (13–21) mmHg, median Child–Turcotte–Pugh (CTP) score was 6 (5–7; CTP stage A: n = 133; B: n = 78; C: n = 18), and median MELD was 11 (9–14) points. Higher PH severity strata was paralleled by stepwise decreases of platelet count (p = 0.002) and increases of CTP and MELD score, as well as prevalence and severity of ascites (all p < 0.001; Table 1).

**Table 1:** Patient characteristics of the overall cohort and patients stratified by portal hypertension severity

Parameter	Overall cohort (n = 229)	HVPG 6–9 mmHg (n = 28)	HVPG 10–19 mmHg (n = 121)	HVPG ≥ 20 mmHg (n = 80)	p value
Age (years)	58 (50–67)	58 (47–67)	59 (50–67)	58 (50–67)	0.807
Sex (M, %)	148 (65)	22 (79)	75 (62)	51 (64)	0.249
Etiology (n, %)					**0.008**
ALD	105 (46)	8 (29)	52 (43)	45 (56)	
Viral	41 (18)	10 (36)	20 (17)	11 (14)	
ALD + Viral	14 (6)	1 (4)	8 (7)	5 (6)	
NASH	23 (10)	3 (11)	19 (16)	1 (1)	
Cholestatic	8 (4)	0 (0)	3 (3)	5 (6)	
Other	38 (17)	6 (21)	19 (16)	13 (16)	
cACLD (n, %)	92 (40)	24 (86)	52 (43)	16 (20)	**< 0.001**
HVPG (mmHg)	18 (13–21)	–	–	–	–
CTP score (points)	6 (5–7)	5 (5–5)	6 (5–7)	7 (6–8)	**< 0.001**
MELD (points)	11 (9–14)	8 (7–11)	10 (9–13)	12 (10–15)	**< 0.001**
Ascites (n, %)					**< 0.001**
None	121 (53)	27 (96)	69 (57)	25 (31)	
Mild	95 (42)	1 (4)	49 (40)	45 (56)	
Severe	13 (6)	0 (0)	3 (3)	10 (13)	
HE (n, %)					0.232
None	182 (80)	26 (93)	96 (79)	60 (75)	
Mild	47 (20)	2 (7)	25 (21)	20 (25)	
Severe	0 (0)	0 (0)	0 (0)	0 (0)	
PLT (G/L)	98 (70–134)	130 (92–162)	98 (70–137)	91 (61–120)	**0.002**
VWF-Ag (%)	269 (214–337)	184 (119–259)	278 (218–337)	291 (240–397)	**< 0.001**
VWF-N (ng/mL)	21.4 (15.5–28.9)	14.8 (9.50–19.5)	21.5 (16.3–28.1)	24.1 (17.8–33.8)	**< 0.001**
VWF-A (ng/mL)	7.93 (4.98–13.7)	5.95 (3.45–7.76)	8.15 (4.84–12.7)	8.98 (5.60–17.3)	**0.013**
VWF-N/-Ag ratio	0.08 (0.07–0.10)	0.09 (0.07–0.09)	0.09 (0.07–0.10)	0.08 (0.07–0.09)	0.712
VWF-Ag/-A ratio	31.3 (19.5–52.3)	33.5 (19.0–44.9)	31.1 (20.9–53.0)	32.2 (18.0–55.1)	0.970
ADAMTS13 activity (%)^a^	106 (84–155)	97 (76–142)	119 (90–167)	97 (81–155)	0.107
VWF-Act (%)^a^	236 (187–309)	170 (130–220)	248 (197–314)	249 (212–348)	**< 0.001**
VWF-Act/-Ag^a^	0.94 (0.84–1.03)	0.97 (0.90–1.11)	0.94 (0.86–1.04)	0.90 (0.79–1.01)	0.107
CRP (mg/dL)	0.25 (0.12–0.56)	0.14 (0.07–0.27)	0.25 (0.11–0.47)	0.40 (0.18–0.69)	**< 0.001**
IL-6 (pg/mL)	7.21 (4.59–12.7)	4.25 (2.78–8.26)	6.83 (4.34–12.4)	8.85 (5.88–18.6)	**< 0.001**
PCT (ng/mL)	0.07 (0.05–0.13)	0.05 (0.03–0.07)	0.08 (0.05–0.13)	0.09 (0.05–0.15)	**< 0.001**
LBP (µg/mL)	6.77 (5.19–8.59)	7.30 (5.64–9.22)	6.91 (5.25–8.59)	6.35 (5.10–8.01)	0.442

Statistical analysis: Kruskal–Wallis and one-way ANOVA were used to compare continuous variables across HVPG strata. Group comparisons of categorical variables were performed using Chi-squared or Fisher’s exact test. p values < 0.05 are indicated in bold

*ADAMTS13* a disintegrin and metalloproteinase with a thrombospondin type 1 motif, member 13, *ALD* alcohol-related liver disease, *cACLD* compensated advanced chronic liver disease, *CTP* Child–Turcotte–Pugh, *HE* hepatic encephalopathy, *HVPG* hepatic venous pressure gradient, *M* male sex, *MELD* model of end stage liver disease, *NASH* non-alcoholic steatohepatitis, *VWF-Ag* von Willebrand factor antigen, *VWF-N* released N-terminal propeptide of VWF, *VWF-A* neoepitope of ADAMTS13-mediated degradation of VWF, *CRP* C-reactive protein, *IL-6* interleukin-6, *PCT* procalcitonin, *LBP* lipopolysaccharide binding protein

^a^Parameter available in 166 patients (HVPG 6–9: n = 23; 10–19: n = 88; ≥ 20 mmHg: n = 55)

### VWF biomarkers and their relation to portal hypertension

**Fig. 1:**
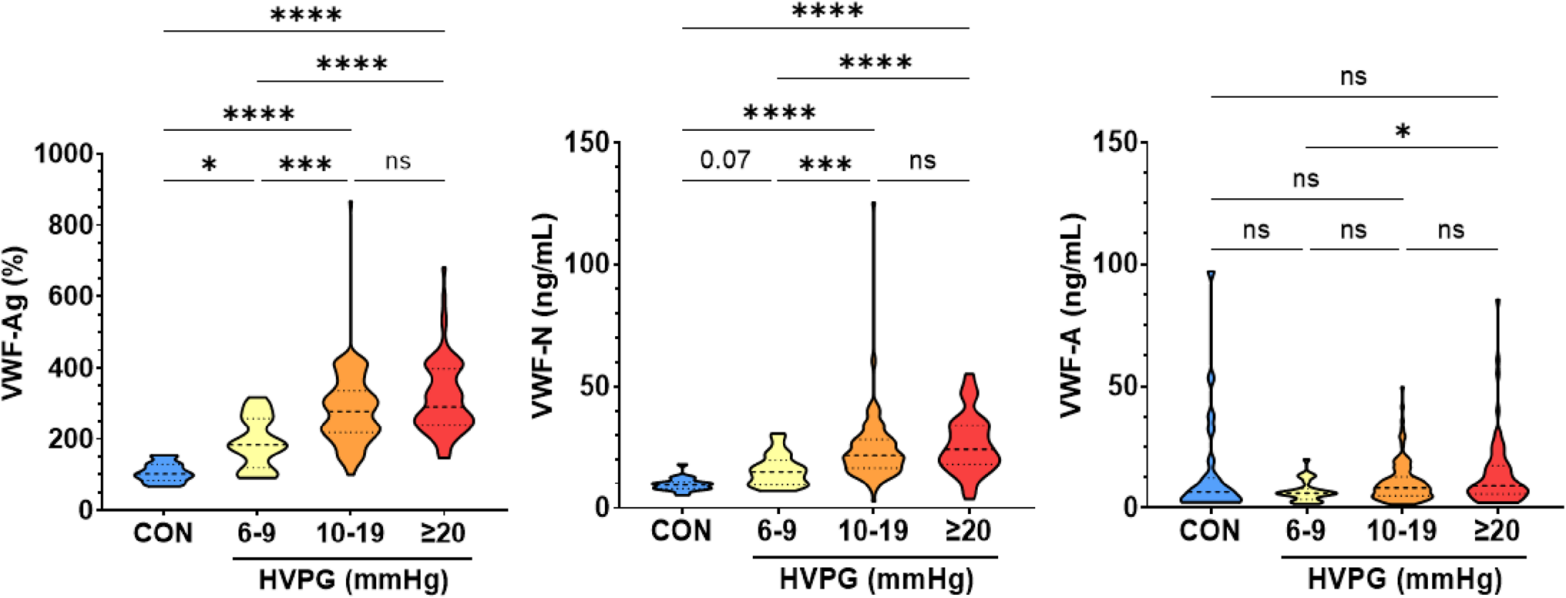
von Willebrand factor (VWF) biomarkers in controls and patients stratified by hepatic venous pressure gradient (HVPG). Statistical analysis: Kruskal–Wallis with Dunn’s multiple comparisons test was used to compare continuous variables. *CON* controls, *HVPG* hepatic venous pressure gradient, *VWF-Ag* von Willebrand factor antigen, *VWF-N* released N-terminal pro-peptide of von Willebrand factor, *VWF-A* neo-epitope of ADAMTS13 mediated degradation of von Willebrand factor

VWF biomarkers were compared between controls (CON) and patients with ACLD stratified by PH severity (HVPG 6–9, 10–19, ≥ 20 mmHg). VWF-Ag levels and VWF-N levels exhibited a stepwise increase between these groups: Median VWF-Ag levels were 102, 184, 278, and 291% (p < 0.001), and median VWF-N levels were 9.44, 14.8, 21.5, and 24.1 ng/mL (p < 0.001). Median VWF-A levels were 6.48, 5.95, 8.15, and 8.98 ng/mL (p = 0.025). VWF-A levels were similar between controls and patients with ACLD, and a statistically significant increase of VWF-A was only observed between patients with an HVPG of 6–9 mmHg and ≥ 20 mmHg (Table 1, Fig. 1).

**Fig. 2:**
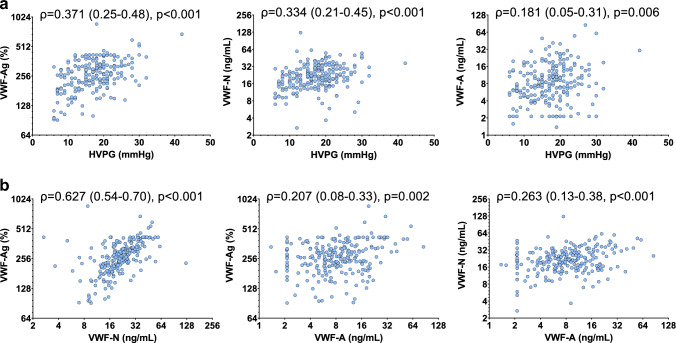
**a** Correlation between von Willebrand factor (VWF) biomarkers and hepatic venous pressure gradient (HVPG). **b** Correlation between von Willebrand factor (VWF) biomarkers. Statistical analysis: Spearman’s correlation coefficients were calculated to assess the association between continuous variables. *HVPG* hepatic venous pressure gradient, *VWF-Ag* von Willebrand factor antigen, *VWF-N* released N-terminal pro-peptide of von Willebrand factor, *VWF-A* neo-epitope of ADAMTS13 mediated degradation of von Willebrand factor

Besides significant positive correlation of VWF-Ag and HVPG (ρ = 0.371, 0.25–0.48; p < 0.001), VWF-N displayed correlation of similar strength with HVPG (ρ = 0.334, 0.21–0.45; p < 0.001). In contrast, VWF-A exhibited a comparatively weak association with HVPG (ρ = 0.181, 0.05–0.31; p = 0.006; Fig. 2A). Next, the relation between VWF-N, VWF-A, and VWF-Ag was assessed to investigate whether VWF propeptide and ADAMTS13-cleaved VWF levels reflect VWF-Ag levels. VWF-N correlated strongly with VWF-Ag (ρ = 0.627, 0.54–0.70; p < 0.001). In comparison, VWF-A was weakly associated with VWF-Ag (ρ = 0.207, 0.08–0.33; p < 0.001) and VWF-N (ρ = 0.263, 0.13–0.38; p < 0.001; Fig. 2B).

### Diagnosis of CSPH by VWF biomarkers in compensated ACLD

**Fig. 3:**
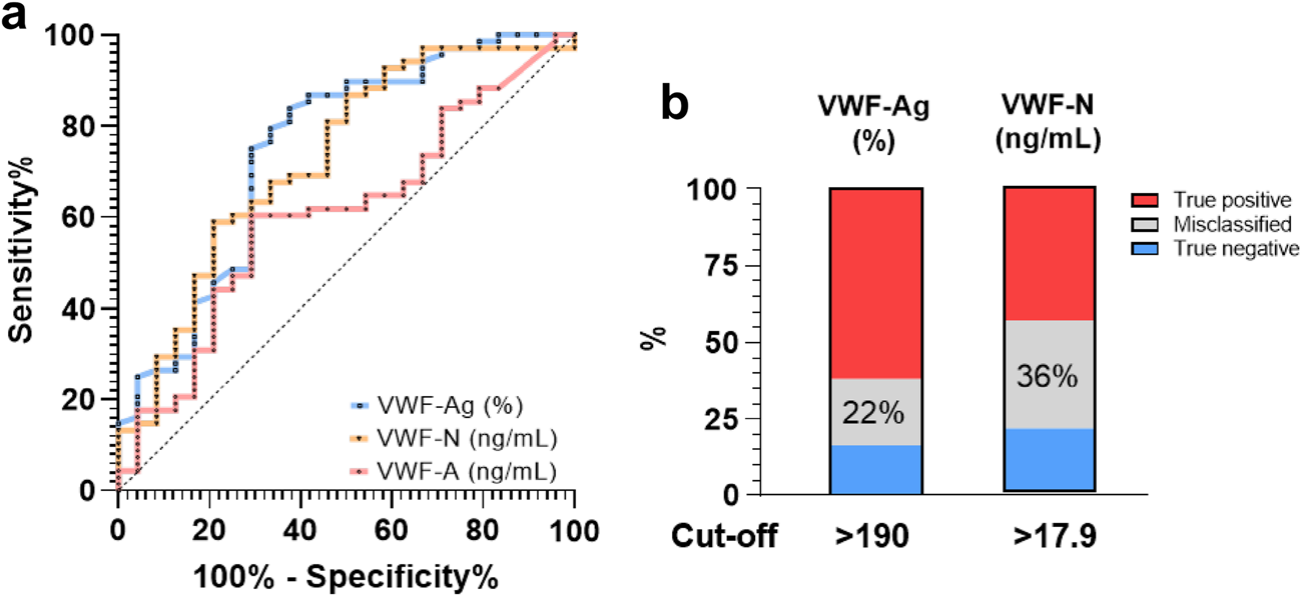
Area-under-the receiver operating characteristics for the prediction of CSPH in compensated ACLD by VWF biomarkers and diagnostic performance of the optimal cut-offs. Statistical analysis: the diagnostic value of biomarkers for prediction of clinically significant portal hypertension (CSPH; i.e., an HVPG ≥ 10 mmHg) was determined by area-under-the-receiver operating characteristics (AUROC). Optimal cut-off levels were determined by Youden’s index (sensitivity + specificity − 1). *VWF-Ag* von Willebrand factor antigen, *VWF-N* released N-terminal pro-peptide of von Willebrand factor, *VWF-A* neo-epitope of ADAMTS13 mediated degradation of von Willebrand factor

Based on previous data suggesting that VWF-Ag has diagnostic value for the presence of CSPH in patients with compensated ACLD (cACLD), we explored whether the VWF propeptide and cleaved VWF displayed a comparable diagnostic performance. VWF-Ag showed an AUROC of 0.748 (0.63–0.87, p < 0.001), VWF-N an AUROC of 0.728 (0.61–0.85, p < 0.001), and VWF-A exhibited an AUROC of 0.607 (0.48–0.74, p = 0.120) (Fig. 3A). The optimal cut-offs (assessed by Youden’s index) were determined as 190% for VWF-Ag, 17.9 ng/mL for VWF-N, while VWF-A was excluded for further analysis. For VWF-Ag, the optimal cutoff yielded a sensitivity of 84% and a specificity of 63%. For VWF-N, the optimal cut-off for CSPH had a sensitivity of 59% and a specificity of 79%. The rate of misclassification (i.e., the sum of false positive and false negative classification when using the optimal cutoff) was 22% for VWF-Ag and 36% for VWF-N (Fig. 3B; Supplementary table S1). Furthermore, we determined highly sensitive and specific cut-offs for diagnosis of CSPH. Cut-offs with ≥ 90% sensitivity were 151% for VWF-Ag (specificity 33%) and 12.3 ng/mL for VWF-N (specificity 42%), while ≥ 90% specificity was achieved at 298% for VWF-Ag (sensitivity 27%) and 22.6 ng/mL for VWF-N (sensitivity 29%). Data on the diagnostic accuracy of the individual cut-offs—including PPV, NPV, PLR, and NLR—are summarized in Supplementary table S2. The performance of VWF biomarkers for prediction of CSPH in the overall cohort is delineated in the Supplementary material.

### VWF processing and its relation to ADAMTS13 activity

Hypothesizing that the turnover of VWF (from release to cleavage) may be reflected by the balance between VWF biomarkers, we explored the relationship PH severity and (i) the balance between VWF release and circulating VWF-Ag (i.e., VWF-N/-Ag ratio) and (ii) the balance between circulating VWF-Ag and ADAMTS13-cleaved VWF (i.e., VWF-Ag/-A ratio).

**Fig. 4:**
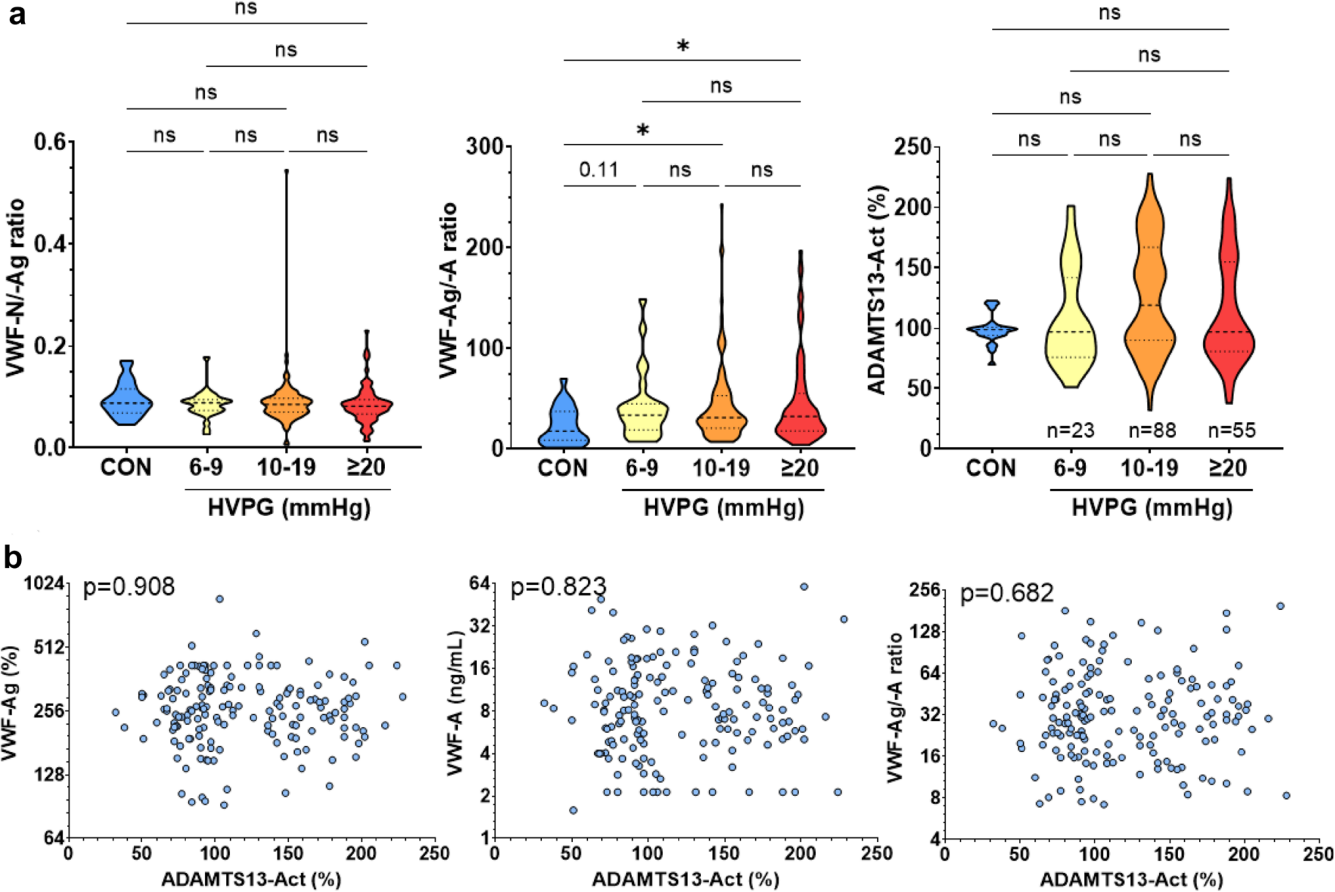
**a** Von Willebrand Factor (VWF) biomarker ratios and ADAMTS13 activity across different stages of portal hypertension. **b** Correlation between ADAMTS13 activity and VWF biomarkers in the circulation. Statistical analysis: Kruskal–Wallis with Dunn’s multiple comparisons test was used to compare continuous variables. Spearman’s correlation coefficients were calculated to assess the association between continuous variables. *ADAMTS13-Act* a disintegrin and metalloproteinase with a thrombospondin type 1 motif, member 13 activity, *VWF-Ag* von Willebrand factor antigen, *VWF-N* released N-terminal pro-peptide of von Willebrand factor, *VWF-A* neo-epitope of ADAMTS13 mediated degradation of von Willebrand factor, *CON* controls, *HVPG* hepatic venous pressure gradient

The VWF-N/-Ag ratio was similar between controls and HVPG strata (p = 0.557). The VWF-Ag/-A ratio, however, increased in patients with ACLD as compared to controls: Median VWF-Ag/-A ratios were 17.9 (5.59–37.2) in controls, 33.5 (19.0–44.9; p = 0.107 vs. CON) in patients with HVPG 6–9 mmHg, 31.1 (20.9–53.0; p = 0.015 vs. CON)with 10–19 mmHg, and 32.2 (18.0–55.1; p = 0.035 vs. CON) with ≥ 20 mmHg. VWF-Ag/-A ratios were similar between HVPG strata, indicating that PH severity was not meaningfully related with a difference in VWF-cleavage (Table 1; Fig. 4A).

Aiming to further investigate the cleavage of VWF in ACLD, ADAMTS13-Act was assessed in a subgroup of 166 patients to investigate whether disease severity and VWF biomarker levels were linked to the activity of the main VWF-cleaving enzyme. Patient characteristics of this subgroup are summarized in Supplementary table S3. ADAMTS13-Act was not associated with disease severity, indicated by similar activity across control individuals and PH strata, as well as patients stratified by CTP stages (p = 0.512) (Table 1; Fig. 4A; Supplementary figure S2). Surprisingly, ADAMTS13-Act neither correlated with VWF-Ag (p = 0.908), VWF-A (p = 0.823), nor the VWF-Ag/-A ratio (p = 0.682; Fig. 4B). Finally, ADAMTS13-Act showed no meaningful correlation with biomarkers of systemic inflammation (Supplementary Figure S3).

### VWF processing and its relation to VWF activity in vitro

VWF-Act was measured in the same subgroup of 166 patients to evaluate whether circulating VWF biomarkers and ADAMTS13-Act relate to the GPIb-binding capacity (i.e., functional properties) of VWF. Furthermore, the VWF-Act/-Ag ratio was calculated to examine whether VWF-Act normalized to VWF-Ag levels exhibited differences across distinct stages of PH severity or was linked to ADAMTS13-Act.

VWF-Act increased between controls and patients with ACLD and was linked to the severity of PH: Median VWF-Act was 108% in controls, 170% in patients with HVPG 6–9 mmHg, 248% with 10–19 mmHg, and 252% with ≥ 20 mmHg (p < 0.001), and also increased when stratifying patients by CTP stages (p < 0.001; Supplementary figure S4). VWF-Act strongly correlated with VWF-Ag (ρ = 0.874, 0.83–0.91), and was—to a lesser extent—linked to VWF-N (ρ = 0.592, 0.48–0.69) and VWF-A levels (ρ = 0.297, 0.19–0.43; all p < 0.001; Supplementary figure S5).

**Fig. 5:**
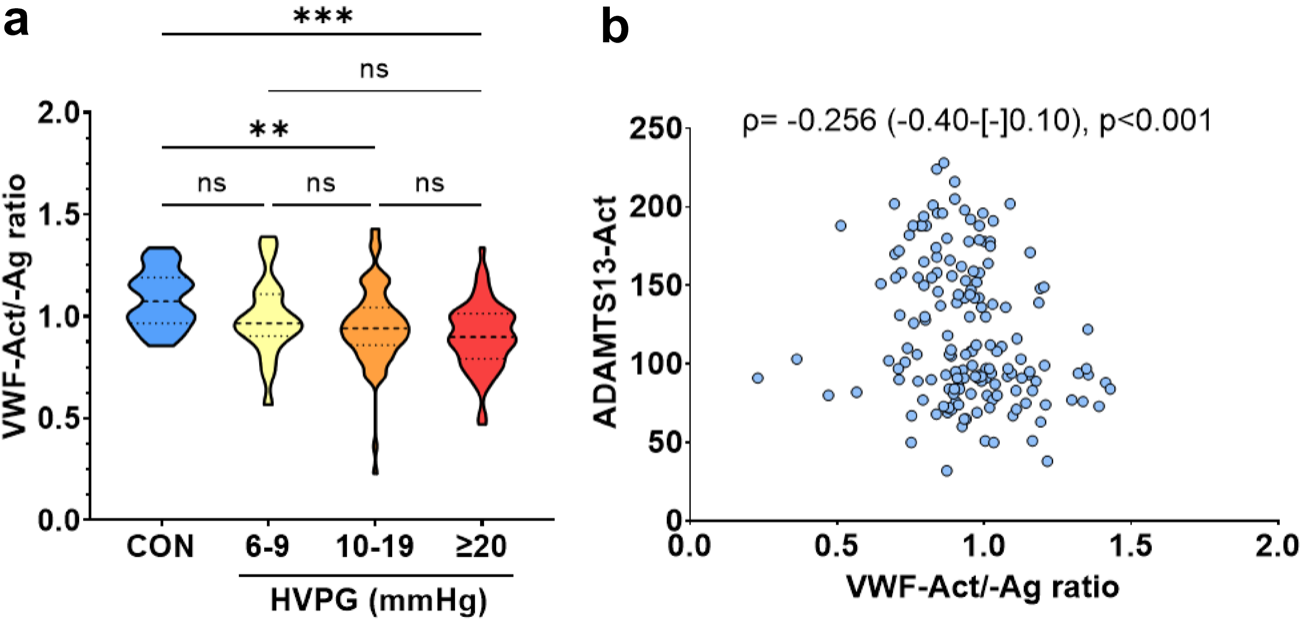
Von Willebrand factor (VWF)-activity/-antigen ratio **a** across different stages of portal hypertension, and **b** its correlation with ADAMTS13-activity. Statistical analysis: Kruskal–Wallis with Dunn’s multiple comparisons test was used to compare continuous variables. Spearman’s correlation coefficients were calculated to assess the association between continuous variables. *VWF-Ag/-Act* von Willebrand factor antigen/activity, *ADAMTS13-Act* a disintegrin and metalloproteinase with a thrombospondin type 1 motif, member 13 activity, *HVPG* hepatic venous pressure gradient, *CON* controls

Importantly, the VWF-Act/-Ag ratio decreased in patients with CSPH as compared to controls: Median VWF-Act/-Ag ratio was 1.07 in controls, 0.97 in patients HVPG 6–9 mmHg (p = 0.329 vs. controls), 0.94 with HVPG 10–19 (p = 0.005 vs. controls), and 0.90 with ≥ 20 mmHg (p < 0.001 vs. controls; adjusted for multiple testing). While VWF-Act as a single parameter was not linked to ADAMTS13-Act (p = 0.135), the VWF-Act/-Ag ratio exhibited a significant negative correlation with ADAMTS13-Act (ρ = – 0.256, – 0.40 to – 0.10, p < 0.001; Table 1; Fig. 5; Supplementary figure S6).

### Predictive value of biomarkers reflecting VWF processing for clinical outcome

Finally, we investigated whether VWF biomarkers or ADAMTS13-Act indicate the incidence of clinical events during the follow-up. Patients with available ADAMTS13-Act (n = 166) had a median transplant-free follow-up period of 27.6 (11.5–40.5) months.

When analyzing predictors of PVT development during follow-up, patients with prior PVT (n = 6) or intake of anticoagulants (n = 5) were excluded. PVT development was recorded in 10 (6%) patients. More specifically, 9 partial and 1 complete PVTs were detected by slice imaging (MRI or CT scan; also see Supplementary table S4). Interestingly, only ADAMTS13-Act was linked to the incidence of PVT (HR per 10%: 0.78, 95% CI 0.63–0.95, p = 0.015; Table 2) indicating that low ADAMTS13-Act was related to a higher risk of PVT.

**Table 2:** Cox proportional hazard models for the risk of portal vein thrombosis

Patient characteristics	Portal vein thrombosis during follow-up (n = 155)					
	Univariate analysis			Multivariate analysis		
	HR	95% CI	p value	HR	95% CI	p value
Age (years)	1.04	0.97–1.10	0.260			
MELD (points)	1.02	0.88–1.18	0.836			
HVPG (mmHg)	1.02	0.91–1.14	0.796			
VWF-Ag (%)	1.04	0.99–1.09	0.126			
VWF-N (ng/mL)	1.02	0.99–1.06	0.229			
VWF-A (ng/mL)	1.03	0.98–1.08	0.323			
ADAMTS13-Act (per 10%)	**0.77**	**0.63–0.95**	**0.014**	**–**	**–**	**–**
CRP (mg/dL)	1.34	0.31–5.79	0.696			

Statistical analysis: Cox proportional hazard models with backwards elimination were performed to assess risk factors for the incidence of portal vein thrombosis during follow-up. p values < 0.05 are indicated in bold

*ADAMTS13* a disintegrin and metalloproteinase with a thrombospondin type 1 motif, member 13, *HVPG* hepatic venous pressure gradient, *MELD* model of end stage liver disease, *VWF*-*Ag* von Willebrand factor antigen, *VWF-N* released N-terminal propeptide of VWF, *VWF-A* neoepitope of ADAMTS13-mediated degradation of VWF, *CRP* C-reactive protein

Furthermore, 62 (37%) events of first/further decompensation or liver-related death during the follow-up period were recorded. HVPG (aHR per mmHg: 1.06, 95% CI 1.02–1.10, p = 0.003) and ADAMTS13-Act (aHR per 10%: 0.93, 95% CI 0.87–0.98, p = 0.013) emerged as independent predictors of this composite endpoint on multivariate analysis adjusting for several other variables such as MELD and CRP levels (Table 3).

**Table 3:** Cox proportional hazard models for the risk of first/further decompensation or liver-related death

Patient characteristics	Decompensation or liver-related death (n = 166)					
	Univariate analysis			Multivariate analysis		
	HR	95% CI	p value	HR	95% CI	p value
Age (years)	1.01	0.98–1.03	0.628			
MELD (points)	**1.07**	**1.011–14**	**0.021**	1.05	0.98–1.12	0.183
HVPG (mmHg)	**1.06**	**1.02–1.11**	**0.003**	**1.06**	**1.02–1.10**	**0.003**
VWF-N (ng/mL)	**1.02**	**1.00–1.03**	**0.022**	1.01	0.99–1.02	0.578
VWF-A (ng/mL)	1.02	0.99–1.04	0.124			
ADAMTS13-Act (per 10%)	**0.93**	**0.87–0.98**	**0.013**	**0.93**	**0.87–0.98**	**0.013**
CRP (mg/dL)	**1.69**	**1.02–2.79**	**0.042**	1.35	0.78–2.35	0.283

Statistical analysis: Cox proportional hazard models with backwards elimination were performed to assess risk factors for the incidence of first/further decompensation or liver-related death during follow-up. p values < 0.05 are indicated in bold

*ADAMTS13* a disintegrin and metalloproteinase with a thrombospondin type 1 motif, member 13, *HVPG* hepatic venous pressure gradient, *MELD* model of end stage liver disease,*VWF-Ag* von Willebrand factor antigen, *VWF-N* released N-terminal propeptide of VWF, *VWF-A* neoepitope of ADAMTS13-mediated degradation of VWF, *CRP* C-reactive protein

## Discussion

This study investigated the relationship between VWF-Ag levels, VWF release and processing, and PH in 229 patients with ACLD undergoing HVPG measurement. Furthermore, we aimed to determine the relationship between ADAMTS13-Act and disease severity, VWF turnover, and VWF activity in a subgroup of 166 patients.

Previous studies indicated a link between elevated VWF levels and fundamental pathological features of cirrhosis such as endothelial dysfunction and PH; moreover, it constitutes a key feature of the hypercoagulability accompanying liver disease progression. Briefly, sinusoidal endothelial dysfunction contributes to the dysregulation of the hepatic vascular tone (promoting PH) and the activation of HSCs (promoting fibrogenesis) [21, 22]. Furthermore, cirrhosis is accompanied by a fragile hemostatic equilibrium due to the simultaneous dysregulation of pro- and anti-hemostatic factors [23]. To this end, it was suggested that elevated levels of VWF counterbalance the reduced numbers and impaired function of platelets [15].

We investigated whether VWF biomarkers reflecting distinct stages of VWF turnover are similarly indicative of the degree of PH in patients with ACLD, since these biomarkers had not been studied simultaneously before. In accordance with previous work assessing VWF-Ag [10, 11] and VWF-N/-A [24], all VWF biomarkers significantly correlated with HVPG in our study. However, VWF-A only exhibited a very weak association with HVPG, whereas VWF-Ag and VWF-N displayed numerically stronger correlations. We acknowledge that our study did not account for multiple comparisons in regard to related VWF biomarkers. Notably, the correlation coefficient for VWF-Ag was considerably higher in the study by Ferlitsch et al. (ρ = 0.69 vs. ρ = 0.37 in our study) [11], which has been conducted at our center before the start of the recruitment period of the present study. This may be explained by the higher percentage of compensated patients (66% vs. 40%) as well as patients without CSPH (26% vs. 12%) in the previous study. Concordantly, a systematic review displayed varying correlations between VWF-Ag and HVPG throughout studies with different patient characteristics [25]. The correlation coefficients between HVPG and VWF-N/-A were also higher in a previous study (ρ = 0.53 vs. ρ = 0.33 and ρ = 0.43 vs. ρ = 0.18 in our study, respectively) [24]. Again, the observed difference to our study may be explained by PH and liver disease severity, as the aforementioned study included patients with HVPG below 6 mmHg (i.e., without PH) [24]. The AUROCs for the detection of CSPH were, however, comparable to our overall cohort [11, 14, 24, 26]. In patients with cACLD, VWF-Ag and VWF-N had the best diagnostic performance for the non-invasive assessment of CSPH in cACLD. While we acknowledge that the sample size of patients with cACLD was rather small in our study, this information is of high clinical relevance, as the diagnosis of CSPH has profound therapeutic implications following Baveno VII [27], as it indicates non-selective betablocker therapy to prevent first hepatic decompensation. Since HVPG measurement is (minimally) invasive and requires considerable resources and expertise, the decision to start NSBB therapy is based on non-invasive tests for estimating the probability of CSPH, with VWF and the derived VITRO score [28] being one of the most promising diagnostic tests [7].

The relative contributions of increases in VWF release and potential decreases in VWF clearance to elevated VWF levels in patients with ACLD are poorly understood [29]. The elevation of both VWF-Ag and VWF-N as well as their strong correlation may be interpreted as evidence of increased release, rather than decreased clearance. In this context, our study is limited by not providing any intrahepatic readouts (e.g., histological data) linking VWF biomarker levels to intrahepatic endothelial dysfunction. Previous data from patients with hepatitis B, however, demonstrated that histological VWF expression in the liver correlated with PH and disease severity [30]. We cannot rule-out that VWF and its propeptide, VWF-N, are cleared by via the same mechanism, which may serve as an alternative explanation for their close interrelation. Macrophages are considered the most relevant cell type for VWF clearance, however, hepatocytes and endothelial cells also play a role in the elimination of VWF from the circulation via lectin and scavenger receptors [31]. The highest amount of VWF seems to be cleared via the liver and spleen. Since ACLD profoundly impacts the functions of all mentioned cell types/organs, it remains to be elucidated, if and to what extent VWF and VWF-N clearance is impacted by ACLD [32]. The ratio between VWF-Ag and VWF-N did not correlate with HVPG in our cohort of clinically stable patients with ACLD and was even comparable to liver-healthy controls. This observation may indicate higher ‘chronic’ release of VWF with increasing PH in patients with ACLD, thus, being different to conditions associated with ‘acute’ VWF release such as sepsis that may be accompanied by a different balance between VWF release (i.e., propeptide) and circulating VWF-Ag [33].

Increases of VWF-A levels weakly correlated with those of VWF-Ag and VWF-N (as well as HVPG), which would indicate that ADAMTS13-mediated turnover tends to follow the concentration of its substrate. However, liver-healthy controls had similar VWF-A levels as compared to patients in our study, indicating a very loose interrelationship. Interestingly, we found that the VWF-Ag/-A ratio decreased in patients with CSPH as compared to liver-healthy controls, which may point towards decreased VWF cleavage. Aiming to further investigate VWF cleavage in clinically stable ACLD, we analyzed ADAMTS13-Act in a large subgroup of the study cohort, given the limited knowledge whether ADAMTS13-Act is related to VWF cleavage, VWF activity, or PH and disease severity [17]. Surprisingly, ADAMTS13-Act was not related to VWF biomarkers or the VWF-Ag/-A ratio, and also similar between different stages of PH and liver dysfunction in our cohort of clinically stable ACLD patients and liver-healthy controls, which add important data to the controversy on ADAMTS13-Act in ACLD. A previous study reported a decrease of ADAMTS13-Act in cirrhotic patients (n = 90) with CTP stages B and C (but not CTP-A) [34] and similar results were reported among ACLD patients with severe impairment of liver dysfunction [35, 36]. Conversely, Lisman et al. exhibited an increase of ADAMTS13-Act in patients with CTP-B, but not CTP-A or CTP-C, in a cohort of 54 patients with ACLD, as compared to healthy individuals [15]. These inconsistent results may be explained by limited sample sizes and differences related to methodology in these studies. Other studies suggested that patient selection has a relevant effect on the relation between liver disease severity, ADAMTS13-Act, and VWF multimer size. High molecular weight VWF multimer levels did not correlate with ADAMTS13-Act in healthy individuals and patients with stable ACLD but displayed significant indirect correlation in patients with acute decompensation [37]. Similarly, ADAMTS13-Act was significantly decreased in patients with overt infections or systemic inflammatory response syndrome, as compared to CTP-A patients [38] and ADAMTS13 antigen levels exhibited stepwise decline in patients with acute decompensation and acute-on-chronic liver failure, as compared to controls and stable cirrhosis [35]. These observations might be clinically relevant as low ADAMTS13-Act was reported in patients with portal vein thrombosis (PVT) [39] and patients with acute decompensation reportedly exhibit a higher prevalence of PVT [40]. In our cohort of patients with stable cirrhosis (i.e. absence of acute decompensation or infections), that marks a difference to other cohorts investigating the prognostic value of VWF e.g. in acute-on-chronic liver failure [41, 42], we found that systemic inflammation increased with PH severity—as reported previously in this cohort [18]—but was not linked to ADAMTS13-Act. These results indicate that low-grade systemic inflammation—which is typical for patients with clinically stable ACLD—is not meaningfully connected with ADAMTS13-Act.

Considering previous work on the decreased proportion of HMW VWF multimers in cirrhotic patients as compared to healthy controls (related to a decreased collagen binding capacity of VWF) [15], we assessed VWF-Act by an in vitro GPIb-binding assay and normalized VWF-Act to circulating VWF-Ag levels (VWF-Act/-Ag ratio). VWF-Act strongly reflected VWF-Ag levels, and importantly, the VWF-Act/-Ag ratio correlated negatively with ADAMTS13-Act. The VWF-Act/-Ag ratio was similar across HVPG strata in our study cohort; however, it was significantly lower in patients with CSPH as compared to controls, which could relate to a functional impairment of VWF-Ag. Nevertheless, it remains to be elucidated how the simultaneous observation of (i) reduced ADAMTS13-related cleavage of VWF (as suggested by the increase of the VWF-Ag/-A ratio as compared to controls) and (ii) the decline in VWF functionality (as suggested by decrease of the VWF-Act/-Ag ratio as compared to controls) is explained. One possibility is the increased cleavage of VWF by other proteases. However, our study cannot provide evidence for/against this hypothesis due to the lack of information on VWF multimer sizes and other VWF-cleaving enzymes.

Finally, we evaluated the association between VWF-related biomarkers as well as ADAMTS13-Act and PVT as well as hepatic decompensation or liver-related death. Notably, ADAMTS13-Act was predictive of PVT development. This is in line with previous case–control [39] studies and a small longitudinal analysis [43], while contrasting the findings of a larger cohort study [44], which questioned the relevance of laboratory tests of coagulation as indicators of PVT risk. Accordingly, further studies evaluating the role of ADAMTS13-Act in this context are warranted.

Interestingly, ADAMTS13-Act associated with hepatic decompensation and liver-related death in an analysis adjusted for HVPG, while no such independent associations were observed for VWF-related biomarkers. Although Reuken and colleagues [38] have previously reported on the association between low ADAMTS13-Act and decreased survival, our study provides novel information in clinically stable outpatients, as the study by Reuken et al. also included patients with acute decompensation and infection. Since ADAMTS13-Act was closely linked to systemic inflammation—i.e., a well-established determinant of prognosis [20, 45]—in the latter study, it may have confounded the association between ADAMTS13-Act and outcome. In contrast, patients with acute decompensation/infection were excluded from our study and further adjustment for CRP did not alter the association between ADAMTS13-Act and hepatic decompensation.

While the independent association between ADAMTS13-Act and adverse clinical events provides evidence for another pathophysiological mechanism contributing to disease progression, it remains unclear whether ADAMTS13-Act is simply a prognostic biomarker or potentially even causatively involved. Regarding the latter point, it may be hypothesized that decreased ADAMTS13-Act is associated with complications via a higher fraction of presumably more procoagulant VWF and macro-/microvascular thrombosis [46], which is in line with the indirect correlation with the VWF-Act/-Ag ratio that was observed in our study. Notably, we abstained from analyzing the prognostic value of ratios between VWF-related biomarkers and ADAMTS13, as the prognostic role of VWF-Ag has been thoroughly investigated [10, 12] and the calculation of ratios between coagulation proteins has several limitations [47, 48]. Thus, we believe that our data may serve as an important stimulus for future research aiming to understand the hemostatic equilibrium in ACLD.

In summary, our study demonstrates that VWF-Ag levels and its propeptide, VWF-N, are better surrogates of PH in patients with ACLD as compared to ADAMTS13-cleaved VWF-A. ADAMTS13-Act is not related to disease severity and circulating VWF-A under clinically stable conditions. While VWF-Ag levels strongly reflect collagen binding capacity in vitro, VWF-Act is disproportionally high in patients with low ADAMTS13-Act, possibly resulting in a procoagulant state. Interestingly, low ADAMTS13-Act was independently linked to PVT development and disease progression. Further studies should investigate a potential causative involvement.

### Supplementary Information

Below is the link to the electronic supplementary material.

Supplementary file1 (DOCX 523 KB)

## Data Availability

Data are available at reasonable request to the corresponding author.

## Abbreviations

ADAMTS13: A disintegrin and metalloproteinase with a thrombospondin type 1 motif, member 13
ADAMTS13-Act: ADAMTS13 activity
ALD: Alcohol-related liver disease
AUROC: Area-under-the-receiver operating characteristics
c/dACLD: Compensated/decompensated advanced chronic liver disease
CON: Controls
CRP: C-reactive protein
CSPH: Clinically significant portal hypertension
CTP: Child–Turcotte–Pugh
HSC: Hepatic stellate cells
HVPG: Hepatic venous pressure gradient
IL-6: Interleukin-6
LBP: Lipopolysaccharide binding protein
LSEC: Liver sinusoidal endothelial cells
MELD: Model of end-stage liver disease
PCT: Procalcitonin
PH: Portal hypertension
PLR/NLR: Positive and negative likelihood ratios
PPV/NPV: Positive and negative predictive values
TIPS: Transjugular intrahepatic portosystemic shunt
VWF: Von Willebrand factor
VWF-Act: VWF activity
VWF-Ag: VWF antigen
