# Supplementary material for: Von Willebrand factor processing in patients with advanced chronic liver disease and its relation to portal hypertension and clinical outcome
Source: Hepatol Int. 2023 Aug 21;17(6):1532–44. doi: 10.1007/s12072-023-10577-y (PMC10661794; doi:10.1007/s12072-023-10577-y)
Supplement: Supplementary file 1 — Supplementary file1 (DOCX 523 KB) [file 12072_2023_10577_MOESM1_ESM.docx]

**SUPPLEMENTARY MATERIAL**

**Title: Von Willebrand Factor processing in patients with advanced chronic liver disease and its relation to portal hypertension and clinical outcome**

**Table of contents**

[Supplementary methods 2](#_Toc120619180)

[Measurement of VWF, VWF-N and VWF-A 2](#_Toc120619181)

[Statistical analysis 2](#_Toc120619182)

[Supplementary results 3](#_Toc120619183)

[Prediction of clinically significant portal hypertension in the overall cohort 3](#_Toc120619184)

[Supplementary tables 4](#_Toc120619185)

[Supplementary figures 7](#_Toc120619186)

# Supplementary methods

## Measurement of VWF, VWF-N and VWF-A

A disintegrin and metalloproteinase with a thrombospondin type 1 motif, member 13 (ADAMTS13)-processed VWF (VWF-A; i.e., neoepitope of ADAMTS13-mediated degradation of VWF) and VWF propeptide (VWF-N; i.e., released N-terminal propeptide of VWF) levels were measured in EDTA plasma samples by competitive enzyme-linked immunosorbent assay (ELISA). Briefly, 96-well streptavidin coated plates (Roche Diagnostics, Mannheim, Germany) were incubated for 30 min at 20°C with a biotinylated synthetic peptide. Standard peptide for the calibration curve or pre-diluted sample were added to appropriate wells, followed by monoclonal antibody and incubated overnight 4°C. Horseradish peroxidase-labeled anti-mouse secondary antibody solution was added and incubated for 1 hour at 20°C. Finally, tetramethylbenzinidine (TMB) (cat.438OH, Kem-En-Tec Diagnostics, Taastrup, Denmark) was added and incubated for 15 minutes at 20°C. All the above incubation steps were performed in darkness and included shaking at 300 rpm. After each incubation step, the plate was washed five times in washing buffer (20 mM Tris, 50 mM NaCl, pH 7.2). The TMB reaction was stopped by adding 0.18 M H2SO4 as stopping solution and measured at 450 nm with 650 nm as reference. A calibration curve was plotted using a 4-parametric mathematical fit model.

# Supplementary results

*Prediction of clinically significant portal hypertension in the overall cohort*

The diagnostic value for prediction of CSPH in the overall cohort was determined. VWF-Ag showed an AUROC of 0.804 (0.72-0.89, p<0.001), VWF-N an AUROC of 0.768 (0.68-0.86, p<0.001), and VWF-A exhibited an AUROC of 0.658 (0.56-0.76, p<0.001) **(Supplementary figure-S7)**. The optimal cut-offs (assessed by Youden’s index) were determined as 207% for VWF‑Ag, 17.6 ng/mL for VWF-N, and 6.78 ng/mL for VWF-A. For VWF-Ag, the optimal cutoff yielded a sensitivity of 84% and a specificity of 68%. For VWF-N, the optimal cut-off for CSPH had a sensitivity of 73% and a specificity of 75%, while for VWF-A, the sensitivity and specificity were 64% and 71%, respectively. The rate of misclassification (i.e., the sum of false positive and false negative classification when using the optimal cutoff) was 19% for VWF-Ag, 27% for VWF-N, and 35% for VWF-A, respectively **(Supplementary figure-S7; Supplementary table-S1)**. Furthermore, we determined highly sensitive and specific cut-offs for the diagnosis of CSPH. Cut-offs with ≥90% sensitivity were 181% for VWF-Ag (specificity 43%), 13.3 ng/mL for VWF-N (specificity 43%), and 2.84 ng/mL for VWF-A (specificity 18%), while ≥90% specificity was achieved at 298% for VWF-Ag (sensitivity 45%), 26.0 ng/mL for VWF-N (sensitivity 38%), and 13.5 ng/mL for VWF-A (sensitivity 28%). Data on the diagnostic accuracy of the individual cut-offs – including PPV, NPV, PLR, and NLR – are summarized in **Supplementary table-S2**.

# Supplementary tables

**Supplementary table-S1. False-positive, false positive, true positive and true negative rates for optimal VWF biomarker cut-offs to diagnose clinically significant portal hypertension (CSPH).**

| **cACLD (n=92)** | | | |
| --- | --- | --- | --- |
| **Parameter** | **Predicted condition** | **True condition** | |
| **VWF-Ag** | **> 190 %** | **HVPG ≥10mmHg (n=68)** | **HVPG 6-9mmHg (n=24)** |
|  | Positive | 57 (62 %) | 9 (10 %) |
|  | Negative | 11 (12 %) | 15 (16 %) |
| **VWF-N** | **> 17.9 ng/mL** | **HVPG ≥10mmHg (n=68)** | **HVPG 6-9mmHg (n=24)** |
|  | Positive | 40 (44 %) | 5 (5 %) |
|  | Negative | 28 (30 %) | 19 (21 %) |
| **Overall cohort (n=229)** | | | |
| **Parameter** | **Predicted condition** | **True condition** | |
| **VWF-Ag** | **> 207 %** | **HVPG ≥10mmHg (n=201)** | **HVPG 6-9mmHg (n=28)** |
|  | Positive | 168 (73%) | 9 (4%) |
|  | Negative | 33 (15%) | 19 (8%) |
| **VWF-N** | **> 17.6 ng/mL** | **HVPG ≥10mmHg (n=201)** | **HVPG 6-9mmHg (n=28)** |
|  | Positive | 147 (64%) | 7 (3%) |
|  | Negative | 54 (24%) | 21 (9%) |
| **VWF-A** | **> 6.78 ng/mL** | **HVPG ≥10mmHg (n=201)** | **HVPG 6-9mmHg (n=28)** |
|  | Positive | 129 (56%) | 8 (3%) |
|  | Negative | 72 (32%) | 20 (9%) |

Abbreviations: (cACLD) compensated advanced chronic liver disease; (HVPG) hepatic venous pressure gradient; (VWF-Ag) Von Willebrand Factor antigen; (VWF-N) Released N-terminal pro-peptide of von Willebrand factor; (VWF-A) Neo-epitope of ADAMTS13 mediated degradation of von Willebrand factor

**Supplementary table-S2. Area-under-the-receiver operating characteristics of von Willebrand Factor (VWF) biomarkers and test performance for diagnosis of clinically significant portal hypertension (CSPH).**

| **cACLD (n=92)** | | | | | | | | | | |
| --- | --- | --- | --- | --- | --- | --- | --- | --- | --- | --- |
|  | **AUROC** | **95%CI** | **P-value** | **Cut-off** | **SENS** | **SPEC** | **PPV** | **NPV** | **PLR** | **NLR** |
| VWF-Ag (%) | 0.748 | 0.63-0.87 | <0.001 | Opt:>190  Sens:>151  Spec:>298 | 0.84  0.91  0.27 | 0.63  0.33  0.92 | 0.86  0.79  0.90 | 0.58  0.57  0.31 | 2.24  1.37  3.19 | 0.26  0.26  0.80 |
| VWF-N (ng/mL) | 0.728 | 0.61-0.85 | <0.001 | Opt:>17.9  Sens:>12.3  Spec:>22.6 | 0.59  0.91  0.29 | 0.79  0.42  0.92 | 0.89  0.82  0.91 | 0.40  0.63  0.31 | 2.83  1.56  3.54 | 0.52  0.21  0.77 |
| VWF-A (ng/mL) | 0.607 | 0.48-0.74 | 0.120 | --- | --- | --- | --- | --- | --- | --- |
| **Overall cohort (n=229)** | | | | | | | | | | |
|  | **AUROC** | **95%CI** | **P-value** | **Cut-off** | **SENS** | **SPEC** | **PPV** | **NPV** | **PLR** | **NLR** |
| VWF-Ag (%) | 0.804 | 0.72-0.89 | <0.001 | Opt:>207  Sens:>181  Spec:>298 | 0.84  0.90  0.45 | 0.68  0.43  0.93 | 0.95  0.92  0.98 | 0.37  0.38  0.19 | 2.60  1.58  6.38 | 0.24  0.23  0.59 |
| VWF-N (ng/mL) | 0.768 | 0.68-0.86 | <0.001 | Opt:>17.6  Sens:>13.3  Spec:>26.0 | 0.73  0.90  0.38 | 0.75  0.43  0.93 | 0.95  0.92  0.97 | 0.28  0.38  0.17 | 2.92  1.58  5.32 | 0.36  0.23  0.67 |
| VWF-A (ng/mL) | 0.658 | 0.56-0.76 | <0.001 | Opt:>6.78  Sens:>2.84  Spec:>13.5 | 0.64  0.90  0.28 | 0.71  0.18  0.93 | 0.94  0.89  0.97 | 0.22  0.20  0.15 | 2.24  1.10  3.93 | 0.50  0.56  0.78 |

Abbreviations: (cACLD) compensated advanced chronic liver disease; (AUROC) area-under-the-receiver operating characteristic; (SENS) sensitivity, (SPEC) specificity; (PPV/NPV) positive/negative predictive value; (PLR/NLR) positive/negative likelihood ratio; (VWF-Ag) Von Willebrand Factor antigen; (VWF-N) released N-terminal propeptide of VWF; (VWF-A) neoepitope of ADAMTS13-mediated degradation of VWF

**Supplementary table-S3. Characteristics of patient subgroups with and without ADAMTS13 activity and VWF activity.**

| **Parameter** | **ADAMTS13 available**  **(n=166)** | **ADAMTS13 not available**  **(n=63)** | **P-value** |
| --- | --- | --- | --- |
| Age (years) | 59 (51-67) | 58 (49-67) | 0.252 |
| Sex (M, %) | 155 (69) | 33 (52) | **0.017** |
| Etiology (n, %)   - ALD - Viral - ALD + Viral - NASH - Cholestatic - Other | 81 (49) 24 (15) 10 (6) 16 (10) 7 (4) 28 (17) | 24 (38) 17 (27) 4 (6) 7 (11) 1 (2) 10 (16) | 0.289 |
| cACLD (n, %) | 65 (39) | 28 (43) | 0.610 |
| HVPG (mmHg) | 17 (12-20) | 19 (13-22) | 0.141 |
| CTP score (points) | 6 (5-7) | 6 (5-8) | 0.449 |
| MELD (points) | 11 (9-14) | 11 (9-14) | 0.667 |
| Varices (n, %)   - None - Small - Large - (Unknown) | 56 (35) 44 (28) 60 (37) 6 | 23 (37) 17 (27) 23 (36) 0 | 0.978 |
| Ascites (n, %)   - None - Mild - Severe | 82 (49) 74 (45) 10 (6) | 39 (62) 21 (33) 3 (5) | 0.238 |
| HE (n, %)   - None - Mild - Severe | 133 (80) 33 (20) 0 (0) | 49 (78) 14 (22) 0 (0) | 0.262 |
| PLT (G/L) | 99 (70-133) | 95 (69-139) | 0.751 |
| VWF-Ag (%) | 262 (210-325) | 298 (231-373) | 0.069 |
| VWF-N (ng/mL) | 20.6 (15.3-29.3) | 23.1 (17.6-28.8) | 0.238 |
| VWF-A (ng/mL) | 8.08 (5.02-13.5) | 7.82 (4.80-14.1) | 0.647 |
| VWF-N/-Ag ratio | 0.08 (0.07-0.10) | 0.08 (0.07-0.10) | 0.649 |
| VWF-Ag/-A ratio | 30.9 (19.1-50.1) | 36.0 (20.5-60.7) | 0.218 |

Statistical Analysis: Student’s t-test or Mann-Whitney-U-test was used to compare continuous variables. Group comparisons of categorical variables were performed using Chi-squared or Fisher’s Exact test. P-values <0.05 are indicated in bold. Abbreviations: (ADAMTS13) a disintegrin and metalloproteinase with a thrombospondin type 1 motif, member 13; (ALD) alcohol-related liver disease; (cACLD) compensated advanced chronic liver disease; (CTP) Child-Turcotte-Pugh; (HE) hepatic encephalopathy; (HVPG) hepatic venous pressure gradient; (M) male sex; (MELD) Model of End Stage Liver Disease; (NASH) non-alcoholic steatohepatitis; (VWF-Ag) Von Willebrand Factor antigen; (VWF-N) released N-terminal propeptide of VWF; (VWF-A) neoepitope of ADAMTS13-mediated degradation of VWF

**Supplementary table-S4. Classification and imaging modalities of portal venous thrombosis (PVT) recorded during the follow-up period.**

| **Number** | **Classification** | **Imaging technique** |
| --- | --- | --- |
| #1 | Partial (incl. VMS) | MRI |
| #2 | Partial (incl. lienal vein) | MRI |
| #3 | Partial | CT |
| #4 | Complete | CT |
| #5 | Partial (incl. lienal vein) | CT |
| #6 | Partial | MRI |
| #7 | Partial | MRI |
| #8 | Partial (incl. VMS) | MRI |
| #9 | Partial | CT |
| #10 | Partial | MRI |

Abbreviations: (MRI) magnetic resonance imaging; (CT) computed tomography; (VMS) superior mesenteric vein

# Supplementary figures

**Supplementary Figure-S1. Patient flow chart.**


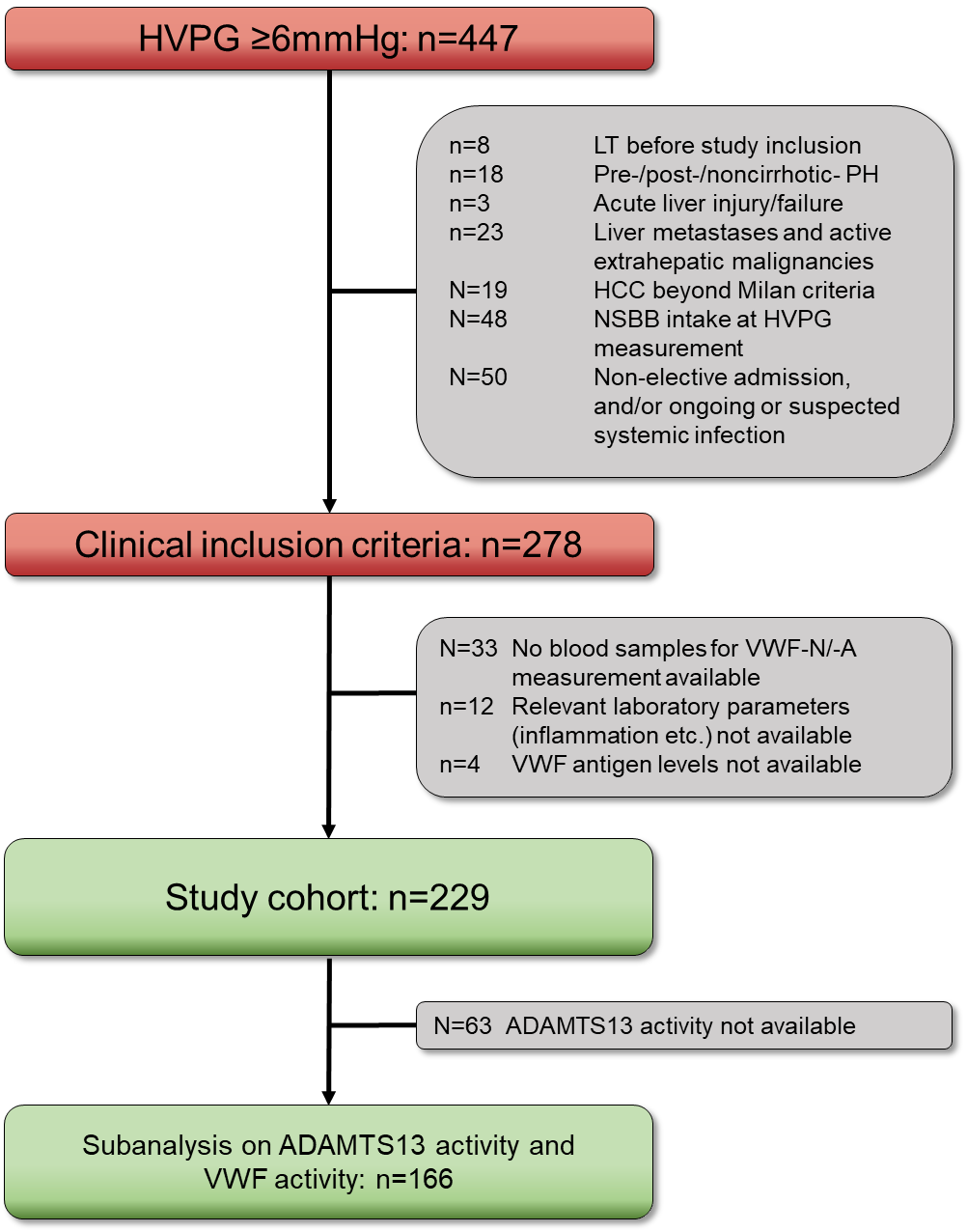


Abbreviations: (HVPG) hepatic venous pressure gradient; (LT) liver transplantation; (PH) portal hypertension; (HCC) hepatocellular carcinoma; (NSBB) non-selective betablocker; (ELF) enhanced liver fibrosis score; (VWF) Von Willebrand Factor; (VWF-N) Released N-terminal pro-peptide of von Willebrand factor; (VWF-A) Neo-epitope of ADAMTS13 mediated degradation of von Willebrand factor; (ADAMTS13) a disintegrin and metalloproteinase with a thrombospondin type 1 motif, member 13

**Supplementary Figure-S2. ADAMTS13 activity in patients stratified by Child-Turcotte-Pugh (CTP) stage.**

Abbreviations: (ADAMTS13) a disintegrin and metalloproteinase with a thrombospondin type 1 motif, member 13; (CTP) Child-Turcotte-Pugh

**Supplementary Figure-S3. Correlation between ADAMTS13 activity and inflammation markers in the systemic circulation.**

Abbreviations: (ADAMTS13) a disintegrin and metalloproteinase with a thrombospondin type 1 motif, member 13; (CRP) C-reactive protein; (IL-6) interleukin-6; (PCT) procalcitonin; (LBP) lipopolysaccharide binding protein

**Supplementary Figure-S4. Von Willebrand Factor activity (VWF-Act) in patients stratified by portal hypertension severity and disease stage.**


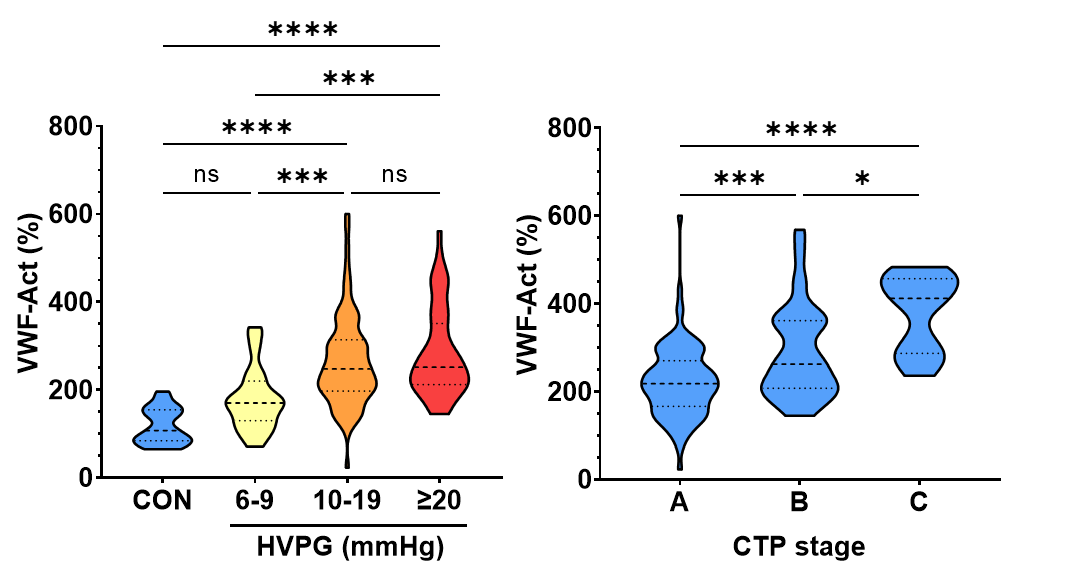


Abbreviations: (CTP) Child-Turcotte-Pugh; (HVPG) hepatic venous pressure gradient; (VWF-Act) Von Willebrand Factor activity

**Supplementary Figure-S5. Correlation between Von Willebrand Factor activity (VWF-Act) and circulating VWF biomarker levels.**


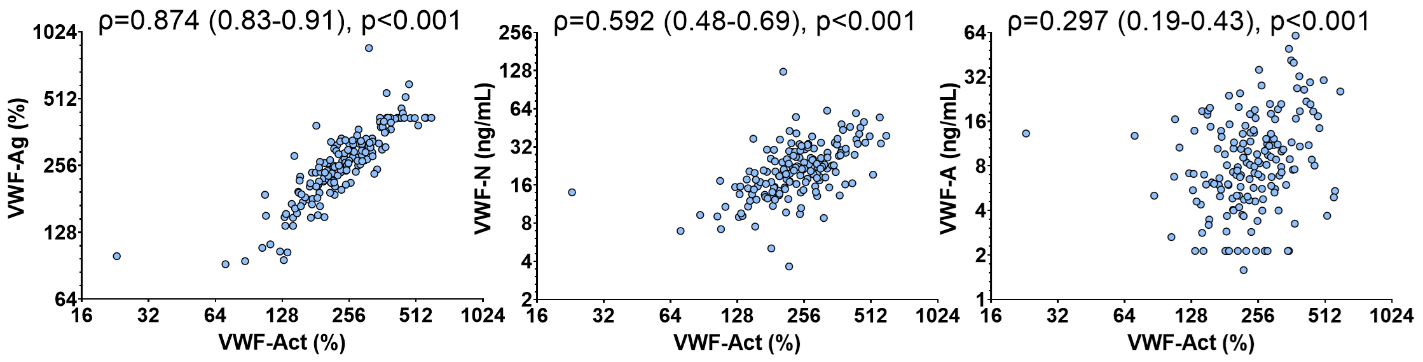


Abbreviations: (CTP) Child-Turcotte-Pugh; (HVPG) hepatic venous pressure gradient; (VWF-Act/-Ag) Von Willebrand Factor activity/antigen

**Supplementary Figure-S6. Correlation between Von Willebrand Factor activity (VWF-Act) and ADAMTS13 activity.**

Abbreviations: (ADAMTS13-Act) a disintegrin and metalloproteinase with a thrombospondin type 1 motif, member 13 activity; (VWF-Act/-Ag) Von Willebrand Factor activity/antigen

**Supplementary Figure-S7. Area-under-the receiver operating characteristics in the *overall cohort* for the prediction of CSPH by VWF biomarkers and diagnostic performance of the optimal cut-offs.**


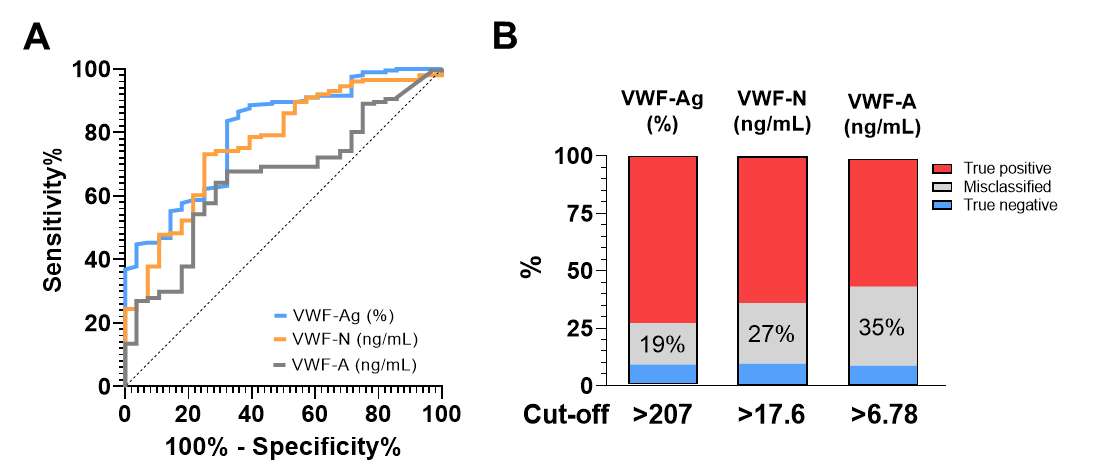


Statistical analysis: The diagnostic value of biomarkers for prediction of clinically significant portal hypertension (CSPH; i.e., an HVPG ≥10 mmHg) was determined by area-under-the-receiver operating characteristics (AUROC). Optimal cut-off levels were determined by Youden’s index (sensitivity + specificity − 1). Abbreviations: (VWF-Ag) Von Willebrand Factor antigen; (VWF-N) Released N-terminal pro-peptide of von Willebrand factor; (VWF-A) Neo-epitope of ADAMTS13 mediated degradation of von Willebrand factor
